# Enabling technology for accelerated discovery of supramolecular materials

**DOI:** 10.1039/d5sc03960f

**Published:** 2025-10-08

**Authors:** C. E. Shields, A. M. Scholes, A. G. Slater

**Affiliations:** a Department of Chemistry and Materials Innovation Factory, University of Liverpool UK anna.slater@liverpool.ac.uk

## Abstract

Organic supramolecular materials, defined by their discrete, modular nature, promise to deliver flexible solutions to pressing challenges, including separations, storage, sensing, and catalysis. The absence of strong metallic or covalent bonding within their solid-state structures enables fine-tuning and post-synthetic processing to tailor properties towards specific applications. However, their production suffers from poor reproducibility, scalability, and sustainability; as a result, translation of these materials from the lab to real-world situations is rare. In this perspective, we discuss how the field stands to benefit from the emergence of ‘enabling technologies’, such as high-throughput screening (HTS), automation, and flow chemistry. We summarise recent advances and consider the opportunities these technologies present for the accelerated discovery, optimisation, and translation of supramolecular materials.

## Abstract 2

Enabling technology such as high-throughput screening, automation, and flow chemistry can accelerate the discovery, optimisation, and translation of supramolecular materials.

## Graphical abstract

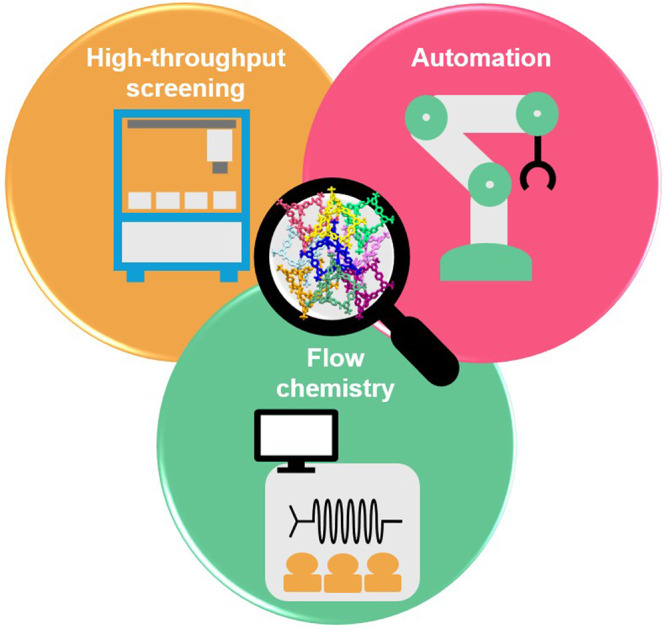

## 1 Introduction

Supramolecular materials consist of discrete organic molecules held together by non-covalent interactions, including hydrogen bonding,^1–3^ halogen bonding,^4–6^ and pi–pi interactions.^7,8^ These structural characteristics distinguish them from extended framework solids, such as covalent and metal–organic frameworks (COFs and MOFs),^9–11^ and give rise to many distinct challenges and opportunities. While MOFs and COFs are well-established materials with many desirable properties, such as excellent thermal and chemical stability and high surface areas,^12–15^ molecular solids can provide solutions for areas where extended framework solids are less suited. For example, the use of discrete and modular building blocks means that supramolecular materials have improved solubility in organic solvents compared to MOFs/COFs, allowing them to be processed into different forms such as mixed membranes and films.^16–20^ The lack of strong bonding between subunits within supramolecular materials can enable flexible and adaptive behaviour that is desirable for fluorescence and sensing,^21–24^ industrially relevant separations,^25–27^ and catalysis without precious metals.^28^ Furthermore, even small modifications to molecular structure can greatly alter the properties of the bulk material, meaning that simple precursors can be fine-tuned to give rise to materials with a diverse range of potential applications. Such precise control over structure has been applied to address challenging molecular separations,^29^ for example, fluorinated from non-fluorinated gases,^30^ and hydrogen from deuterium.^31^

However, the same properties that give rise to this flexible and tailorable nature also result in difficulties in maintaining reproducible and scalable production. The formation of functional supramolecular materials can be separated into two stages: first synthesis, then post-processing such as crystallisation and/or post-synthetic modification. Both stages present chances for control over structure and properties, while also introducing additional challenges that must be addressed for translation from the lab to real-world use.

The discrete components of supramolecular materials, such as organic cages and macrocycles,^32–36^ are often synthesised using the principles of dynamic covalent chemistry (DCC), which uses reversible covalent reactions to allow for error correction and enable self-assembly under thermodynamic control.^37–39^ Such molecular self-assembly allows complex products to be isolated from simple precursors in straightforward, high-yielding reactions, avoiding time-consuming multi-step synthetic routes. Furthermore, careful manipulation of the assembly process, *e.g.* through choice of solvent, temperature, or concentration, can be used to access unlikely, energetically disfavoured species.^40^ However, self-assembly can sometimes result in the formation of completely unexpected or unwanted species.^41–43^ As such, the reaction environment for reversible reactions is important: small changes can influence the equilibrium between the possible species that can be formed. The more complex the reaction mixture, for example when aiming for mixed or self-sorted species, the greater the effect these small changes can have – making it hard to predict or control the process.^44–49^ Consequently, supramolecular synthesis can suffer from poor reproducibility, and extensive time, resources, and energy can be spent trying to understand and optimise the process. Synthetic methods such as high dilution and templating can help form the desired species under DCC,^50–52^ however, this then requires additional reagents and reaction steps, limiting both the scalability and sustainability of the material.

**Fig. 1:**
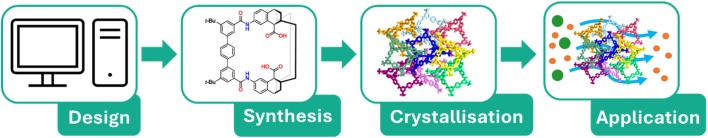
The discovery pipeline for crystalline supramolecular materials. Promising targets are identified, the molecular components are synthesised, then crystallisation yields the material in its final form, suitable for use in its intended application (*e.g.* gas separations or molecular sieving).

When working with supramolecular materials, synthesis of the desired molecule is usually only half of the challenge. Once the molecular components have been obtained, further processing is typically required to obtain the desired material in its final form (Fig. 1). The term ‘molecular materials’ encompasses many sub-classes of material, from amorphous solids to porous liquids.^53–56^ The second half of this perspective will focus on crystalline supramolecular materials, and the difficulties that arise from predicting and controlling their crystallisation. The crystal structure of a material—how the individual molecules arrange themselves in the solid state—strongly influences its bulk properties. For example, molecules may pack closely to give a dense structure, or inefficiently to forms pores.^57,58^ Often, organic molecules can crystallise in either close-packed or porous arrangements depending on the experimental conditions, a phenomenon known as polymorphism. For organic supramolecular materials, polymorphism is often viewed as a hurdle which must be overcome to design a material with a specific function. The resulting structure can arise from a delicate balance of competing intermolecular forces, and it is not always intuitive how this balance will play out. However, polymorphism can also present an exciting possibility: if polymorph selection can be controlled, simple precursors can give rise to diverse structures with distinct properties including pore size or topology, surface area, and selectivity for molecular separations.

As standard batch methods have limitations in terms of throughput, scalability, and environmental control, the specific complexities arising during the formation of supramolecular materials may be addressed through the use of “enabling technologies”. This term encompasses a broad range of techniques, but this perspective will focus on three main areas: high-throughput screening (HTS), flow chemistry, and automation.

HTS enables hundreds, even thousands, of experiments to be conducted in parallel, allowing fast and efficient exploration of, *e.g.*, polymorphism,^59^ or the combinatorial screening of novel precursors.^60^ Flow chemistry refers to reactions that are conducted in a continuously flowing stream,^61^ offering more control over reaction conditions, easier scale-up, and improvements in safety and scalability. Both HTS and flow chemistry can be automated through the integration of robotics, in-line analysis, and software.^62–64^ Commercially-available automation platforms, including liquid and solid handlers, autosamplers, and robotic arms, are becoming more accessible and advanced, although still come with high upfront costs. Many groups are also opting to build their own custom solutions, using standard lab equipment or 3-D printed components controlled by scripts.^65–67^

The rising use of enabling technology in materials chemistry has already delivered improvements such as faster exploration of process space, better reproducibility, higher yield and selectivity, and more.^68–73^ The reversible processes used to form supramolecular materials, from precursor synthesis to crystallisation, stand to benefit enormously from such approaches.

**Fig. 2:**
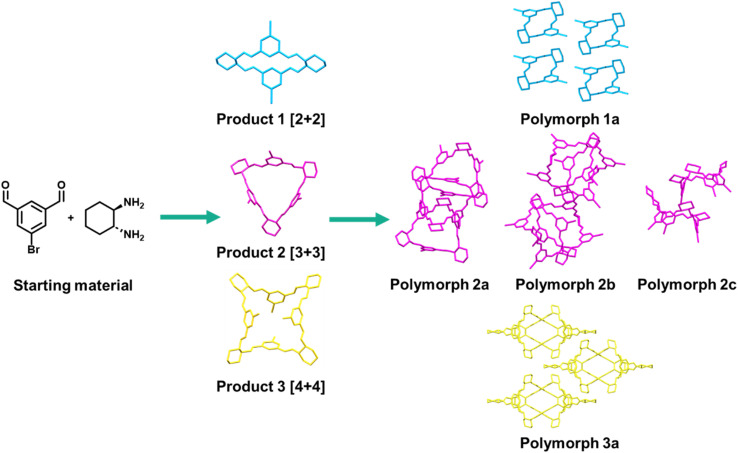
Reversible supramolecular chemistry can result in a range of products, highlighted here with the observed outcomes of bromo-isotrianglimine synthesis and crystallisation, isolated through manual screening of limited parameter space.^45,78^ Further factors that can influence similar reversible reactions include temperature, time, concentration, pH, water content, and salts/additives.

For example, imine macrocycles formed from isophthalaldehyde derivatives represent a supramolecular system where the discovery, synthesis, and post-synthesis modification steps could be improved by enabling technology. The reversible synthesis of isotrianglimines results predominantly in a mixture of [2 + 2] and [3 + 3] macrocycles that are in equilibrium with each other in solution.^74^ Methods of shifting this equilibrium to one product are limited,^75,76^ however recent work by Scholes *et al.* varied crystallisation conditions to obtain the [2 + 2] and [3 + 3] macrocycles separately, as well as a larger [4 + 4] macrocycle that was not present in the solution equilibrium mixture.^45^ Furthermore, the [3 + 3] macrocycles form multiple polymorphic structures which can also assemble in the solid state in different supramolecular motifs (Fig. 2).^77^ The range of products from two simple precursors exemplifies the sensitivity of the reaction to environmental factors and the breadth of potential materials possible, both in this specific case and for the wide range of supramolecular systems formed from reversible processes. Exploring the vast potential chemical and process space requires both reproducible and scalable approaches; attempting this manually would require prohibitive amounts of time and material, and risk high rates of false positives and negatives *via* poor control of reaction environment. As such, this example highlights the need for strategies that allow for the fine control of processes in materials chemistry, whilst considering scalability, sustainability, and time from the outset.

In this perspective, we will highlight key examples of uses of enabling technology for the discovery of supramolecular materials, under two headings: solution-phase synthesis and crystallisation. We will summarise the advances in this area, including our recent work, discussing advantages and potential pitfalls, and proposing routes to the efficient, scalable, sustainable discovery and manufacture of these materials.

## 2 Solution phase synthesis

### 2.1 High-throughput screening for precursor synthesis

**Fig. 3:**
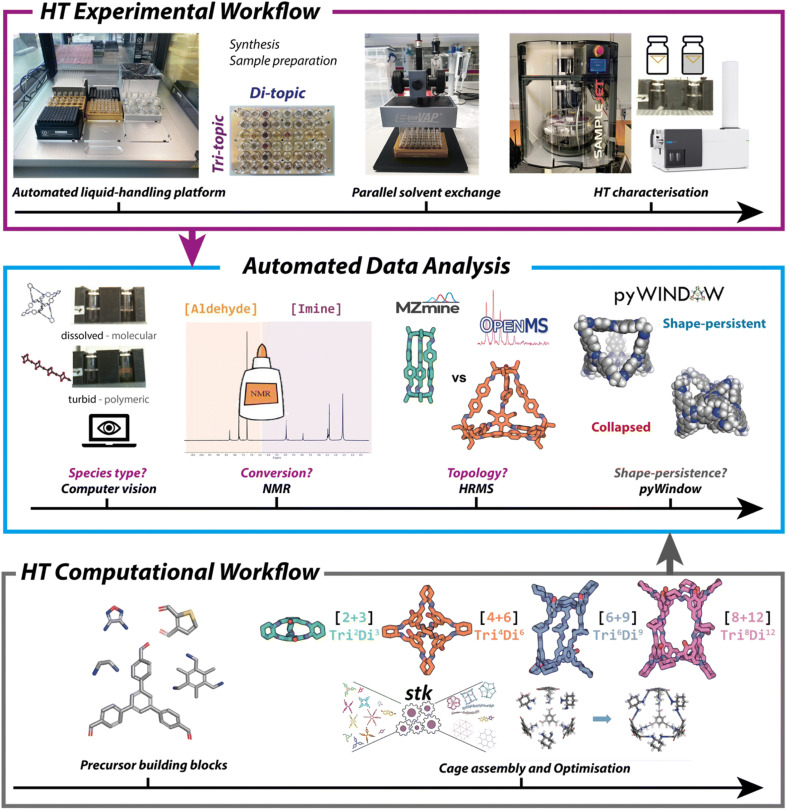
An automated, high-throughput workflow for the synthesis, analysis, and property prediction of POCs. Image reproduced with permission from ref. 60.

A limiting factor for the discovery of new materials is the time it can take to synthesise and analyse the molecular components, especially where there is a large chemical space to explore. High-throughput screening methods can be used to test multiple conditions simultaneously, but the high cost of commercial platforms can be a barrier to entry. Furthermore, for HTS to be beneficial there needs to be an equally high-throughput analytical method in place to evaluate reaction outcomes, otherwise analysis becomes a process bottleneck. Building on initial work by Greenaway *et al.*, which reported a high-throughput experimental workflow for the synthesis of porous organic cages (POCs) and catenanes,^79^ Basford *et al.* demonstrated the potential of a low-cost automated liquid handling for combinatorial screening of POC precursors.^60^ They used 55 commercially available and easily synthesised aldehydes and amines to screen 366 imine condensation reactions. Automation was applied to streamline the analysis and: (i) identify the type of species formed, using computer vision to assess solubility and classify a reaction as successful (soluble, discrete product) or unsuccessful (turbid solution, polymer); (ii) evaluate reaction conversion, using Python code to analyse the aldehyde and imine regions of ^1^H NMR spectra; and (iii) determine the topological outcome, using high resolution mass spectrometry (HRMS) to identify expected cage masses predicted through use of a freely available Python package, pyWindow (Fig. 3).^80^ The workflow resulted in a 350-fold reduction in the time required for data analysis compared with manual methods, as well as reducing the barrier to entry by using low-cost components. More recently, Basford *et al.* also applied a similar workflow to the discovery of metal organic cages,^81^ and the integration of computational and experimental workflows has been reviewed by Greenaway and Jelfs.^82^

Although HTS has many advantages when exploring wide chemical spaces, there are some limitations. For example, a compound that is challenging to synthesise by hand is likely to be at least as challenging for an automated system to make, and potentially completely out of reach if complex handling steps are required. For supramolecular synthesis, where intramolecular forces are important in the assembly of the desired product, the reaction environment is extremely important, and this sensitivity can cause batch-to-batch variation. A subtle change in reaction temperature, local concentration, or stirring can result in a significant impact on the self-assembly process. As such, promising precursors can be identified by computational methods, but can prove hard to synthesise, low-yielding, or only form within narrow process windows; these can be missed with a high-throughput screening strategy. A final obstacle of HTS is the challenge of translating screening results to larger scale production of substrates: complete re-optimisation of conditions can be required when moving from a well-plate or small vial to a larger batch vessel.

### 2.2 Flow chemistry for fine control and scale-up

**Fig. 4:**
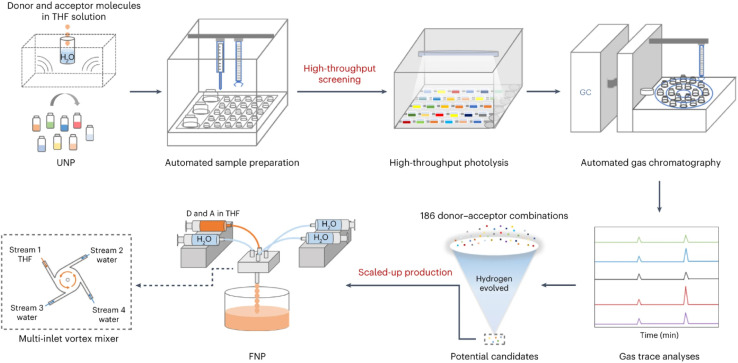
High-throughput workflow for the screening of novel organic small-molecule photocatalysts. Image reproduced with permission from ref. 83.

While the application of HTS as a stand-alone technique has its limitations, combining small-scale batch screening with continuous flow chemistry has proven to be a successful approach for the synthesis of novel supramolecular materials. Flow chemistry provides routes to scale up a reaction by either running a process continually or by increasing the number of reactors, therefore keeping the reaction parameters consistent and reducing re-optimisation requirements. A combined HTS-flow approach was recently used for the discovery of molecular nanojunction photocatalysts (Fig. 4).^83^ A combinatorial library of 186 donor–acceptor hybrids was generated by conducting small-scale Hantzsch pyridine condensation reactions followed by ultrasonic nanoprecipitation. A HT photocatalysis screening workflow identified the most active donor–acceptor combinations, which were then produced on a larger scale using a semi-continuous flow nanoprecipitation process (FNP). The approach outlined here found a cyano-substituted pyridine-based material, MTPA-CA:CNP147, with an exceptionally high sacrificial hydrogen evolution rate of 330 mmol h^−1^ g^−1^. The use of enabling technologies meant that the entire process, from initial screening and property testing to scale up, was completed in just a few weeks.

In addition to facilitating scale-up, flow chemistry can provide enhanced control over experimental variables (temperature, flow rate, reaction volume, better heat/mass transfer), and therefore the reaction environment, compared to batch.^61^ Integration of automation and inline analysis with flow processes provides real-time data and reaction monitoring, offering the opportunity to rapidly screen more conditions for each reaction.

Such process control is particularly useful for self-assembled species that are usually carried out at a milligram scale due to their sensitivity to reaction parameters, such as self-assembled metal–ligand architectures.^84^ For example, the formation of molecular knots, and other topologically complex structures, are often low yielding due to the formation of multiple unwanted side products.^39,85^ Recently, Du/Padgham *et al.* used the enhanced control of flow chemistry to increase the molecular throughput of molecular helicates and knots, improving both reproducibility and scalability (*e.g.*, for one example, increasing the throughput from 18.3 mg h^−1^ to 282 mg h^−1^).^86^ Transferring the process to flow also allowed the synthesis of a zinc(ii) helicate that is inaccessible *via* standard methods. This highlights how new technologies, such as flow, can be used to access distinct products from those formed in batch, especially for systems that rely on reversible processes to form them, producing enough material to enable application screening. However, continuous flow is typically operated in a serial manner, losing the advantages of the highly parallelised reaction screening of HTS.

**Fig. 5:**
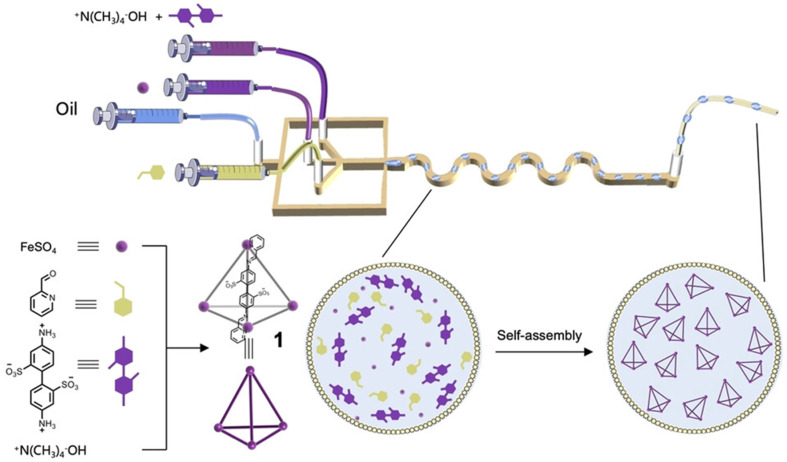
Self-assembly of metallacages in microdroplets using microfluidic technology. Image reproduced with permission from ref. 88.

High-throughput droplet reactors combine the advantages of both approaches. An effective approach to control the reaction environment is to form microdroplets as miniaturised reaction vessels (Fig. 5).^87^ Each microdroplet is equivalent to a ‘reactor’, which, due to the volume confinement, has increased mass-heat transfer, mixing, surface area-to-volume ratio, and surface effects compared to the bulk solution. Lin *et al.* used this approach for the efficient self-assembly of metallacages in microdroplets,^88^ reporting five different metallacages that self-assembled in minutes and in quantitative yields, in contrast to the batch reactions which required hours to complete. For example, one metallacage was produced in a yield of 95% in 14 minutes using the microdroplet approach, compared to the reported 83% yield after 20 hours in batch.^89^ The metallacages were subsequently used as catalysts for ring–opening reactions: within the microdroplets, the catalytic reaction efficiency of the metallacages was considerably improved due to spatial confinement.

For supramolecular systems where there is a need to favour the thermodynamic and/or discrete product over a kinetic and/or polymeric product, synthetic methods such as high-dilution or slow additions of the reactants are often used. Therefore, when scaling a target material, these methods require an excess amount of solvent which is both economically and environmentally unfavourable. For these systems it can be beneficial to implement alternative methods of synthesis. For example, flow chemistry has been used to synthesise imine POCs, CC1 and CC3 with reduced reaction times and reduced solvent use, removing the need for high-dilution methods, whilst maintaining high yields and purity.^90^ Transferring the reaction to flow reduced the synthesis time from three and five days in batch, respectively, to 10 minutes with yields of 93% and 95% (allowing for a throughput of 0.35 g h^−1^ and 0.5 g h^−1^). Both cages were isolated by direct precipitation of the reaction mixture into hexane to give the crystallised materials. The significant reduction in reaction time is due to the ability to heat DCM at 100 °C by using a back pressure regulator to pressurise the system alongside improved mixing and heat transfer. The batch and flow methods for CC3 synthesis were recently compared in terms of their ‘macrocyclisation environmental impact’, concluding that the transfer to flow improved this metric nearly 50-fold.^91^ Other reversible reactions that rely on high-dilution methods may also benefit from such approaches where controlled mixing provided in flow allows the use of higher concentrations, providing a more sustainable method of scale-up – although it must be noted that a full life cycle analysis is required before the sustainability of a process can be determined, including energy use, waste generation, and feedstocks.^92,93^

### 2.3 Mechanochemical methods

An alternative low-solvent method of synthesising POCs is twin-screw extrusion (TSE), which is a form of mechanochemistry where the need for solvent can be removed or greatly reduced through the grinding of neat reagents.^94,95^ TSE can carry out mechanical synthesis of supramolecular materials in a continuous process that can be scaled to values of kg h^−1^.^96^ Mechanochemistry has been used for macrocycles,^97,98^ metal organic cages,^99,100^ rotaxanes,^101,102^ and co-crystals,^103,104^ and can be advantageous for porous materials as it removes the need for desolvation which can affect a material's porosity. TSE has been used to synthesise CC3 for large scale production, and showed improved throughput, reduced reaction time, and lower solvent use compared to both batch and flow methods (Table 1).^105^ The resultant crude cage material was amorphous by PXRD analysis and showed reduced gas uptake compared to expected values. However, the crude material could be purified post-synthesis by using Soxhlet extraction to obtain crystalline ‘technical grade’ CC3, which had comparable gas uptake to pure CC3.

**Table 1:** Comparison of the solvent volume and reaction time required to make 5 g of CC3 by different synthetic methods, and overall rate of formation, reproduced from ref. 105

	Solvent volume (mL)	Reaction time (h)	Rate of formation (g h^−1^)
Batch^106^	154	120	—
Flow^90^	600	10	0.5
TSE^105^	2.96	0.56	8.89

CC3 is just one example where the properties of the resulting material are dependent on its crystallinity, emphasising the importance of post-synthetic processing for the production of supramolecular materials with targeted solid-phase applications. High crystallinity is not always beneficial: it has been reported that amorphous CC3 can have higher porosity than crystalline CC3.^92^ However, while amorphous packing might be better for some applications, it is impossible to control or reproduce, as the arrangement of molecules in the solid state is random. For crystalline supramolecular materials, which are the focus of this Perspective, the molecules instead arrange themselves in a specific, highly ordered fashion. It can therefore be possible to predict and influence the assembly process, enabling fine control over the resulting properties. The following section discusses the challenges faced in controlling supramolecular crystallisation, and how they can be addressed through the application of enabling technologies.

## 3 Crystallisation

Much like the processes that occur during synthesis, the supramolecular assembly that takes place during crystallisation is a delicate balance of intermolecular interactions and even small changes to molecular structure can tip this balance to favour one arrangement over another.^107–109^ These subtle effects are not always intuitive, and therefore it can be difficult, if not impossible, to predict or control how a molecule will crystallise. Crystal engineering strategies provide a classical approach to understand and manipulate the intermolecular interactions that govern crystallisation to target materials for specific functions.^110,111^ While these strategies serve as useful guidelines for the design of molecular crystals, the subtle influence of competing interactions or environmental conditions mean that they are not always successful. Computational methods such as crystal structure prediction (CSP) have emerged as complementary techniques to crystal engineering, and can help direct experimental efforts towards promising molecules, as well as providing further understanding of the intermolecular forces in action.^112–114^

**Fig. 6:**
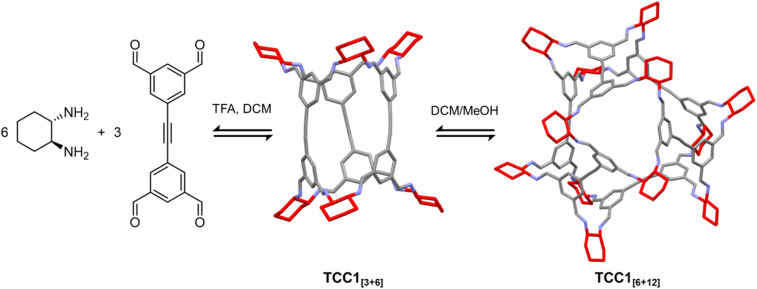
Re-equilibration of imine cage TCC1_[3+6]_ to the larger cage TCC1_[6+12]_ under crystallisation conditions.^47^

A further complication arises for molecules formed by dynamic covalent chemistry, which can undergo further scrambling and self-sorting under crystallisation conditions.^46^ The labile nature of bonding within these structures means that molecular rearrangement can occur upon dissolution in the crystallisation solvent, resulting in serendipitous new structures. For example, during the crystallisation of tubular imine cage TCC1_[3+6]_, formed by the condensation of three equivalents of tetra aldehyde with six equivalents of diamine,^115^ a larger cage species, TCC1_[6+12]_, was isolated unexpectedly (Fig. 6).^47^ Redissolving TCC1_[3+6]_ in chloroform, with methanol or ethanol as an antisolvent, enabled solvent-mediated re-equilibration to the [6 + 12] cage, which crystallised out of solution concomitantly with the expected [3 + 6] cage. Molecular modelling suggested that the energy difference between the two cages is small, and could be overcome by changing the reaction conditions, *i.e.* solvent, equilibration time, and temperature. Similar behaviour was observed by Abet *et al.* when attempting to synthesise two diastereomeric imine cages by the reaction of an aldehyde with either *R*,*R*- or *S*,*S*-cyclohexanediamine.^49^ The products of neither reaction could be identified, but when they were co-crystallised, scrambling of the building blocks led to subsequent formation of a dissymmetric cage with three vertices in the *R*,*R*-configuration and three vertices in the *S*,*S*-configuration. Other examples of molecular rearrangements upon crystallisation include the formation of catenated (interlocked) cages,^116,117^ and interconversion of [2 + 2], [3 + 3], and [4 + 4] isotrianglimines.^45^

The occurrence of molecular scrambling and polymorphism means that obtaining target crystal structures experimentally can be a slow and inefficient process, even with the guidance of crystal engineering and CSP. While these techniques can be helpful for molecular design, high computational cost means they cannot generally account for external factors like choice of solvent, concentration, or temperature, all of which can alter the crystallisation outcome.^107,118,119^ Furthermore, structures with desirable properties, such as porosity, are rarely the thermodynamically favoured form and may only be obtained from specific crystallisation conditions.^113,120–122^ Polymorphs with notably different physical properties are often separated by only a few kJ mol^−1^.^123^ Consequently, the potential search space for molecular crystals is huge, and investigating their complex solid-state landscapes can be a daunting task. Large-scale crystallisation screens are often required to find suitable conditions for target polymorphs, but when performed manually this can be incredibly time-consuming, and interesting materials are missed as a result. Here, too, enabling technologies can help, allowing us to fully explore and take advantage of this vast crystallisation space.

### 3.1 High-throughput crystallisation screening

Automation and HTS enable many experiments to be prepared on a relatively short timescale, which is particularly useful for finding suitable crystallisation conditions or searching for new polymorphs. Typically, powder X-ray diffraction (PXRD) is more amenable to automation than single-crystal XRD (SCXRD) due to the challenges of preparing suitable single crystal samples. For example, Yotsumoto *et al.* recently reported an autonomous robotic PXRD workflow that was able to accurately determine the anatase content of titanium dioxide using just three milligrams of sample.^124^ Robertson and co-workers have also developed methods for in-line PXRD analysis and controlling crystallisation in flow.^125–128^

When combined with crystal structure prediction, HT-PXRD can be a valuable tool for the discovery of new materials. Cui *et al.* demonstrated this combined computation-automation approach with discovery of new structures of well-studied molecules, trimesic acid (TMA) and adamantane-1,3,5,7-tetracarboxylic acid (ADTA).^59^ A CSP study identified stable, previously unknown structures for both materials, including a potentially porous form of TMA. A HT crystallisation screen was then conducted, using a Chemspeed platform to dispense stock solutions of TMA and ADTA into vials, along with an antisolvent. After drying, the powder samples were manually loaded onto the diffractometer, and the resulting PXRD patterns were compared to the CSP data. Once suitable crystallisation conditions had been identified, single crystals were grown and analysed manually to confirm the structures. This approach successfully identified the predicted porous form of TMA. However, only six of out 280 crystallisation experiments yielded the new form, highlighting the need for HT screening.

**Fig. 7:**
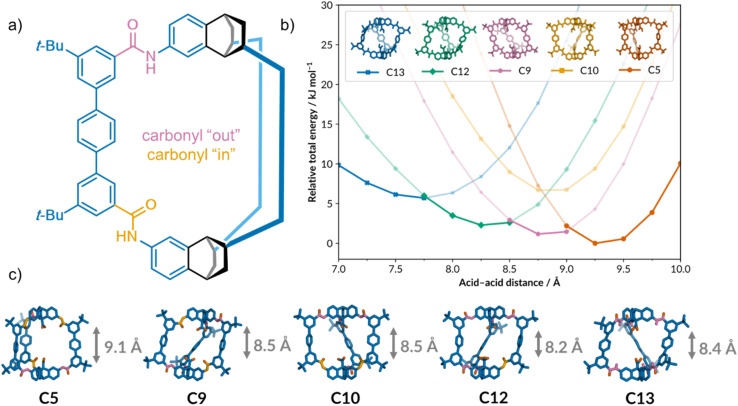
(a) The structure of cage 1, showing the amide carbonyls pointing into and out of the cage cavity. The cage scaffold has been simplified for clarity. (b) The five predicted stable conformers of cage 1 and their relative stabilities over a range of cavity heights. (c) The experimentally obtained crystal structures of the five conformers. Adapted from ref. 129 with permission.

Along with solid-state structure and bulk properties of a material, crystallisation can also give an insight into a molecule's dynamic behaviour in solution. Shields *et al.* recently demonstrated this for a flexible organic cage, which was predicted to exist in multiple conformations due to restricted rotation around the six amide bonds in the cage scaffold (Fig. 7a).^129^ Computational models showed that the cavity height of cage 1 (Fig. 7b) can change depending on the relative orientations of the amide carbonyls, making it an interesting candidate for adaptive guest binding applications. The different cage conformations could not be resolved by NMR, presumably due to fast exchange in solution, so crystallisation was used to identify them. Inexpensive computational calculations identified which of the 13 statistically possible conformations would be stable under ambient conditions. A semi-automated solubility screening and crystallisation protocol was then developed using a commercially available ChemSpeed Swing ISynth robotic solid and liquid dispensing platform to target the predicted conformers. Using this combined computational-HTS approach, all five of the predicted stable conformers of cage 1 (Fig. 7c) were identified, including a potentially porous polymorph.

Using automation and HTS to prepare crystalline samples allows many experimental conditions to be tested rapidly and can greatly accelerate the discovery process. However, as for synthesis, analysis of the resulting crystals then becomes the bottleneck. For synthetic workflows, key analytical techniques can be incorporated fairly easily, using flow cells to enable in-line NMR and IR measurements,^130–132^ or autosamplers to prepare aliquots of the reaction mixture for HPLC-MS analysis.^133^ In contrast, integration of solid-state techniques, including XRD, is very difficult. In general, handling solids has proved a significant challenge for automation, predominantly due to the difficulty of transferring and manipulating solids with different physical properties. Commercially available solid dispensing solutions exist, but are far less advanced than liquid handlers, and often require large amounts of material, which is not practical for materials discovery workflows. Handling solids in flow is notoriously problematic, although solutions to address this issue, such as controlling flow regimes,^126,134^ inline agitation and sonication,^135–137^ and wider-bore tubing and mixers^138,139^ have emerged in recent years.

**Fig. 8:**
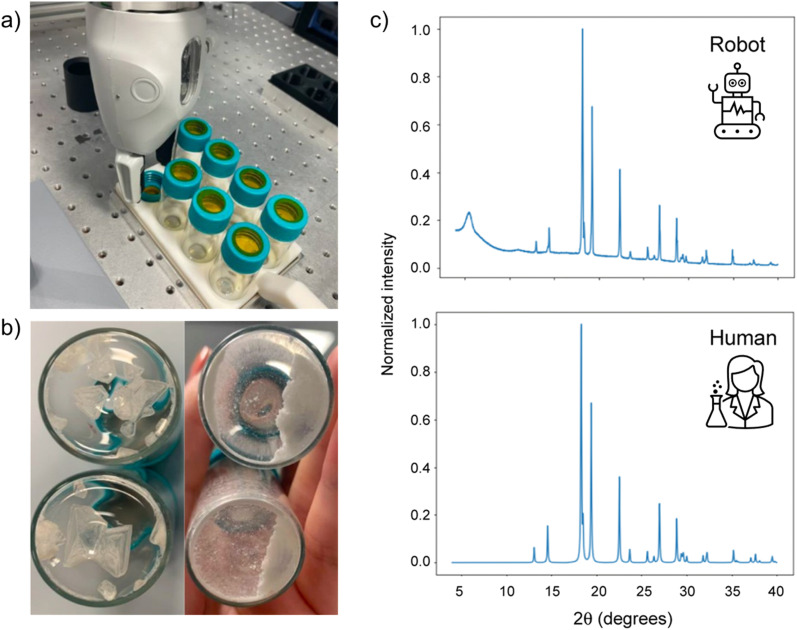
Automated polymorph screening procedure. Crystals are grown in vials capped with Kapton tape to aid solid transfer (a). Samples are ground (b), then the vial caps are removed and analysed by PXRD (c). Adapted from ref. 140 with permission.

To overcome the challenges associated with handling solids in an automated fashion, several groups have turned to custom-built solutions. Lunt *et al.* reported a robotic PXRD workflow using a ChemSpeed Flex platform and a dual-arm robot to prepare crystalline samples (Fig. 8).^140^ Samples were dispensed into vials as stock solutions, then after drying the resulting crystals were ground using a stirrer bar and shaker plate. To minimise solid transfer, the ground samples were analysed directly on the vial caps, using Kapton tape to adhere to the sample, and a 3-D printed holder to load the caps directly onto the diffractometer. A mobile robot transferred the vials between stations, allowing continuous operation with minimal human intervention. The effectiveness of the workflow was demonstrated using highly polymorphic small molecules ROY and benzimidazole but could, in theory, be transferred a materials discovery workflow.

### 3.2 Crystallisation in flow

**Fig. 9:**
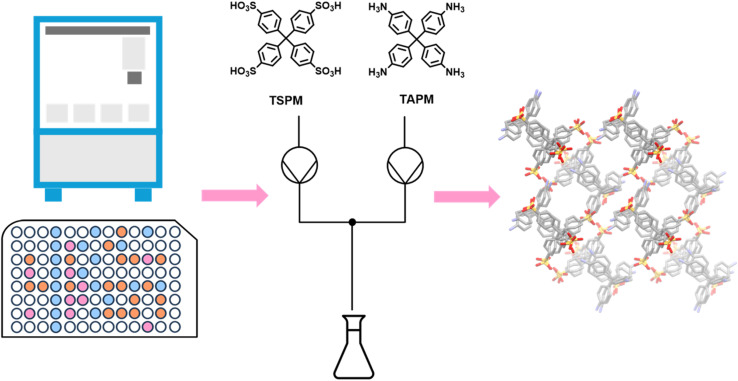
The discovery of a novel crystalline porous organic salt (CPOS-7) using HTS and flow chemistry. Adapted from ref. 141 with permission.

While HTS aims to use as little material as possible, once a particularly interesting polymorph has been found, producing the material on a large scale then becomes the challenge. Here, as for synthesis, combining small-scale screening approaches with continuous flow chemistry can help. O'Shaughnessy *et al.* used HTS to rapidly screen co-crystallisation conditions for crystalline porous organic salts (CPOSs, Fig. 9).^141^ A new CPOS material (CPOS-7) was identified, and suitable single crystals were obtained directly from the screen. However, scaling up the material for function testing while retaining its crystallinity proved difficult. A successful scale-up procedure was developed using flow chemistry to precisely control the mixing of conformers and produced enough highly crystalline CPOS-7 for subsequent gas sorption analysis. CPOS-7 exhibited high stability and a CO_2_ gas uptake of 4.3 mmol g^−1^, making it one of the most porous CPOS materials reported to date.

### 3.3 Crystallisation on the microscale

Despite the benefits automation and flow chemistry have to offer, there are very few examples of their use for the crystallisation of supramolecular materials. Before wide-scale uptake can be expected in the field, there are several key challenges to overcome. Firstly, many of the methods reported use a relatively large amount (several hundred milligrams) of material per screen; for discovery workflows, where material is often scarce, these methods are not practically applicable. To conduct full and thorough exploration of chemical space, smaller scale approaches are needed. There are many examples of small-scale crystallisation in biology and the pharmaceutical industry from which we can take inspiration. For example, Tyler *et al.* reported a method based on the microbatch-under-oil technique commonly used in protein crystallography which enabled the automated preparation of hundreds of crystallisation experiments with just a few milligrams of sample.^142^ The potential of the encapsulated nanodroplet crystallisation (ENaCt) method has been demonstrated by the growth of diffraction-quality single crystals of an ‘uncrystallisable’ agrochemical, dithianon, as well as the discovery of two new structures of renowned polymorphic small molecule, ROY.^142,143^ ENaCt has also been successfully applied to the crystallisation of challenging organic cage-based HOF materials.^144^

**Fig. 10:**
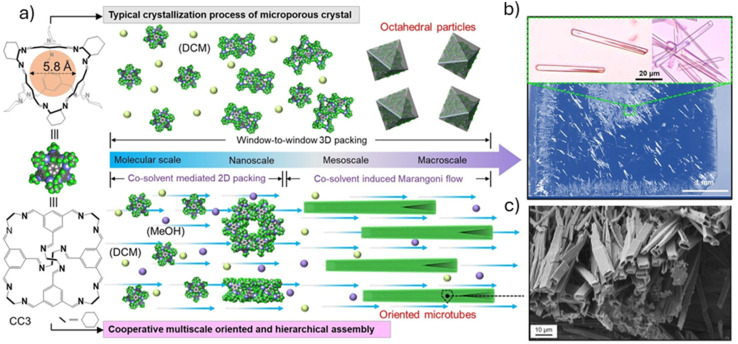
Schematic representation of the standard crystallisation process of CC3 compared to the cooperative control strategy (a). Optical microscopy (b) and SEM (c) images of the crystalline CC3 microtubes. Images reproduced with permission from ref. 145.

Not only do scaled-down crystallisation approaches use less material, but they can also enable fine control over molecular assemblies. For example, Liu *et al.* recently reported a cooperative assembly strategy for the crystallisation of organic cage molecules over multiple length scales (Fig. 10). Injection of a 15 μL droplet of imine cage CC3 in a binary solvent system onto a silicon wafer led to the formation of highly crystalline 1-D cage microtubes. The use of a ‘directing’ solvent, methanol, manipulates the packing of cage molecules towards the tubular structure at the nanoscale, while also introducing a Marangoni flow regime within the droplet at the meso-/macroscale, resulting in elongated crystals with a hierarchical pore structure with aligned pore channels. The approach was then expanded using geometry confinements to engineer fluid flow within the solvent droplets, resulting in the formation of highly oriented 2-D and 3-D cage microtubes.^145^

### 3.4 Automating diffraction

The second major challenge in automating the crystallisation of supramolecular materials is the difficulty in preparing and handling single crystalline samples. As discussed, HT-PXRD is invaluable for narrowing down the search towards promising materials, but single crystal analysis then becomes a bottleneck. The ability to obtain good quality single crystal data is a crucial part of the materials discovery pipeline, elucidating the relationship between structure and function, distinguishing different solvates and polymorphs, and characterising novel materials. However, even growing and analysing suitable single crystals manually can be hugely challenging, if not impossible. Molecular crystalline materials, especially porous molecular crystals, are often very unstable to desolvation due to the relatively fragile supramolecular interactions connecting the framework.^58,108^ Therefore, developing automated methods to prepare and analyse single crystals *in situ* could be a way forward, as exemplified by sophisticated synchrotron beamline set-ups.^146–149^ Electron diffraction (ED) has also gained prominence in the last decade, enabling full structural characterisation in minutes from nanometre-sized crystals.^150^ Although full automation of ED experiments is a long way off, recent advances in data collection and analysis have led to a more widescale uptake,^151–155^ and ED has shown promise for the characterisation of fragile porous materials, crystalline supramolecular polymers,^156^ and structural transitions in flexible crystals.^157^

## 4. Future outlook

While the use of enabling technologies for the discovery of functional supramolecular materials has only just begun to emerge, the potential benefits of their wide-scale uptake are evident. High-throughput screening, flow chemistry, and automation promise to accelerate each stage of the discovery process, from synthesis of molecular building blocks to crystallisation screening and scale-up, offering improvements in safety, productivity, and reproducibility. The use of these technologies enables promising materials to be identified and scaled up efficiently, reducing the barrier from lab-scale to industrial-scale production.

It is evident that the conditions by which a supramolecular material is made can significantly influence the final properties of the material. By using enabling technologies that offer inline analysis, reproducibility, and finer control of parameters, the synthesis-process-structure–function relationship of a material can be better understood. Furthermore, high-throughput screening allows a huge amount of information to be gathered relatively easily, giving more of an insight into these systems than ever before. This information can then be used to guide the next generation of discovery, working towards the intuitive design of materials with a targeted function or property. Although the examples here have focused primarily on porous, crystalline supramolecular materials, such techniques are readily applicable to other materials classes, including those whose structure is both complex and essential for advanced function: *e.g.*, piezoelectrics, non-linear optics, co-crystals, and soft materials.^158–161^

Looking forward, integration of experimental automation with machine learning (ML) and artificial intelligence (AI) stands to advance materials discovery even further.^164,165^ ML approaches have already seen substantial progress in chemical design, planning of synthetic routes, property prediction, and analysis. For example, Turcani *et al.* developed an ML model for predicting cavity size and shape persistence in organic cages.^162^ Dai *et al.* recently reported the autonomous discovery of host-guest assemblies using a heuristic decision maker to screen the synthesis of metal–organic cages and subsequently perform guest-binding titrations.^163^ Optimisation algorithms such as Bayesian optimisation can also be integrated to efficiently explore reaction space and improve yields or reduce environmental impact, for example.^163–166^ The development of robust and reliable ML tools depends upon the curation of large, standardised databases of materials, which automation and HTS platforms are poised to deliver.

Although the outlook is promising, and the field is continually advancing, there are still significant hurdles to overcome before the full potential of these technologies can be reached. The diverse range of synthetic and analytical procedures required in a materials discovery workflow means that automation must be flexible and modular. Because of the rigidity and high-upfront costs of many commercially available platforms, groups are instead opting for modified or custom-built solutions, but this can create more of an entry barrier for chemists looking to develop and operate these systems. Furthermore, standardisation of methods and ensuring reproducibility becomes more difficult, requiring better transparency when reporting results, such as the inclusion of metadata. Addressing these concerns requires not only a solid understanding of chemistry, but also of data science and engineering.^159,160^ As the field of materials discovery shifts to face unprecedented challenges, bridging the gap between these disciplines through additional training and sharing of knowledge becomes increasingly important.

## Author contributions

Conceptualization: Caitlin Shields, Abbie Scholes, Anna Slater; writing – original draft: Abbie Scholes, Caitlin Shields; writing – review and editing: Abbie Scholes, Caitlin Shields, Anna Slater; supervision: Anna Slater; funding acquisition: Anna Slater.

## Conflicts of interest

There are no conflicts to declare.

## Data Availability

No primary research results, software or code have been included and no new data were generated or analysed as part of this review.
